# Prevalence of Anemia in Children with Congestive Heart Failure due to Dilated Cardiomyopathy

**DOI:** 10.1155/2012/452909

**Published:** 2012-11-19

**Authors:** Goetz Christoph Mueller, Emmy Lou Schlueter, Florian Arndt, Ali Dodge-Khatami, Jochen Weil, Thomas S. Mir

**Affiliations:** Department of Pediatric Cardiology, University Heart Center, University Medical Center Hamburg-Eppendorf, Martinistraße 52, 20246 Hamburg, Germany

## Abstract

*Introduction*. Anemia is prevalent in adult heart failure patients and appears to be an independent risk factor for morbidity and mortality. The purpose of this work is to determine the prevalence of anemia in children with heart failure from dilated cardiomyopathy (DCM) and to evaluate its influence on morbidity and mortality. *Methods*. A homogenous group of 58 children with congestive heart failure from DCM was evaluated for heart failure symptoms, appearance of anemia, hospitalization, age of first clinical appearance, necessity of transfusion, and death during medical attendance. Anemic and nonanemic patients were analyzed for differences in age distribution, morbidity, and mortality. *Results*. Anemia was present in 64% of DCM patients. Hospitalization secondary to heart failure was significantly elevated in heart failure patients with anemia (mean 35.1 ± 40.5 versus 9.97 ± 9.65 days per year, *P* < 0.05). However, mortality was not elevated. Significant relations of age and prevalence of anemia or age and severity of anemia did not appear. *Conclusion*. Anemia is prevalent in pediatric patients with congestive heart failure from DCM and appears in all age classes. Hospitalization as a surrogate of morbidity is elevated in heart failure patients developing anemia, but mortality risk did not increase.

## 1. Introduction

Congestive heart failure (CHF) is an important, growing public health problem causing substantial morbidity and mortality [1]. In adults, the most common etiology of heart failure is coronary artery disease [2]. In pediatric patients, congenital heart disease and cardiomyopathy are the most common offenders. 

There is increasing recognition of the high prevalence and importance of comorbidities in congestive heart failure. Anemia plays a unique role, considering the similarities in symptoms and the importance of oxygen carrying capacity in the manifestation of heart failure. In adult patients with heart failure, anemia is prevalent and appears to be an independent risk factor for higher mortality associated with CHF [3]. These data have resulted in the potential therapeutic targeting of anemia. Studies have suggested a clinical benefit with increased exercise tolerance and quality of life from anemia treatment with intravenous iron and erythropoietin [4, 5].

To our knowledge, the prevalence of anemia and its influence on morbidity and mortality are unknown in pediatric patients with congestive heart failure due to dilated cardiomyopathy (DCM). Therapeutic options and their influence on children are not reported.

The purpose of this work is to determine the prevalence of anemia in children with heart failure due to DCM and to evaluate the correlation of heart failure and anemia and their influence on morbidity and mortality. 

## 2. Methods

Patient's data were obtained retrospectively by reviewing of medical records from the Department of Pediatric Cardiology of the University Heart Center Hamburg. The study confirms the principles outlined in the Declaration of Helsinki, and all necessary points were approved by local ethic committee for human research at the Medical Association of Hamburg, Germany. 

Between 1997 and 2007, a study population of 58 patients with drug-treated chronic congestive heart failure due to dilated cardiomyopathy was identified and analyzed in order to investigate a homogenous study population. Dilated cardiomyopathy resulted from myocarditis in five and from Bland-White-Garland-Syndrome in two patients. The remaining share of the study population presented with idiopathic dilated cardiomyopathy. All 58 patients were analyzed retrospectively for appearance of anemia during check-up at the outpatients clinic or at admission to the ward in case of decompensated heart failure. Further on, age of first clinical heart failure symptoms, necessity of transfusion, and death during follow up were analyzed. 

Symptoms of CHF included diaphoresis, tachypnea, breathing with abdominal retraction, respiratory rate, hepatomegaly, and medical treatment were considered. ROSS and NYHA scores were not available in this retrospective study. Morbidity due to heart failure was quantified in days per year of hospitalization in case of decompensated heart failure for inotropic support or adjustment of enteral medications. 

Anemia was defined by hemoglobin levels according to the following age- and gender-specific criteria: 1 day of life (DOL) < 15.2 g/dL, 2–6 DOL < 15 g/dL, 14–23 DOL < 12.7 g/dL, 24–37 DOL < 10.3 g/dL, 40–50 DOL < 9 g/dL, 2–2.5 months < 9.2 g/dL, 3–3.5 months < 9.6 g/dL, 5–7 months < 10.1 g/dL, 8–10 months < 10.5 g/dL, 11–13.5 months < 10.7 g/dL, 1–1.9 years < 11 g/dL, 2–4.9 years < 11.2 g/dL, 7–7.9 years < 11.4 g/dL, 8–11.9 years < 11.6 g/dL, 12–14.9 years male < 12.3 g/dL, female < 11.8 g/dL, 15–17.9 years male < 12.6 g/dL, female < 12 g/dL, > 18 years male < 13.6 g/dL, female < 12 g/dL [6].

Hemoglobin levels were checked at variable times at the outpatient clinic or at admission to the ward at time of decompensated heart failure symptoms. Concerning to age- and gender-specific criteria mentioned above, anemia was diagnosed. Hemoglobin levels under 8 g/dl were used as an indication for blood transfusion. 

Patients with congestive heart failure due to congenital heart disease were not included in this study, because of the heterogeneous nature of the defects and the high prevalence and causal relation of CHF with surgery and postoperative treatment. DCM patients without blood withdrawal or less than three hemoglobin levels were not included because of insufficient data. 

Data sets concomitant to iatrogenic influences like operations, extracardiac life support, or oncological, renal, and hematological diseases were excluded.

Statistical analysis: the data were processed using a standard statistical package (SPSS 15.0, SPSS Inc., Chicago, IL, USA) for Windows software. All data are reported as mean ± standard deviation. *P* values less than 0.05 were considered statistically significant. Continuous variables were compared with a Mann-Whitney-*U*-test. For the correlation of age and severity of anemia, Pearson's correlation coefficients were calculated.

## 3. Results

The study population of 58 pediatric DCM patients was investigated over a followup time of 3.74 +/− 3.69 years for the prevalence of anemia. All patients were on medication for CHF. Three patients had to be excluded because repetitive hemoglobin values were not available. In a group of 55 patients, anemia appeared in 64% of patients during followup. 

The study population was separated into groups of patients with or without development of anemia; general features of these patients are reported in Table 1. 

**Figure 1:**
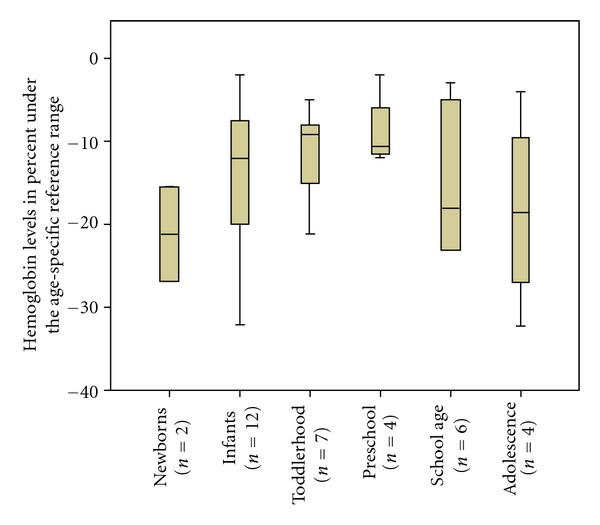
Hemoglobin levels of the analyzed age groups. Boxplots represent median and interquartiles. The whiskers extend to 1.5 times the difference between the first and third quartiles.

To asses if the early onset of disease predicts a higher risk of developing anemia in case of congestive heart failure, anemic and nonanemic patients were compared. Age distribution of first clinical onset of DCM did not differ between the groups (Table 1). In order to analyze if younger age predicts increased severity of anemia, correlation of age at onset of anemia and severity of anemia were calculated. No correlation of age at onset of anemia and severity of anemia could be found (*P* = 0.229). In Figure 1, severity of anemia in percent under the age-specific reference range is presented for different age groups. 

**Figure 2:**
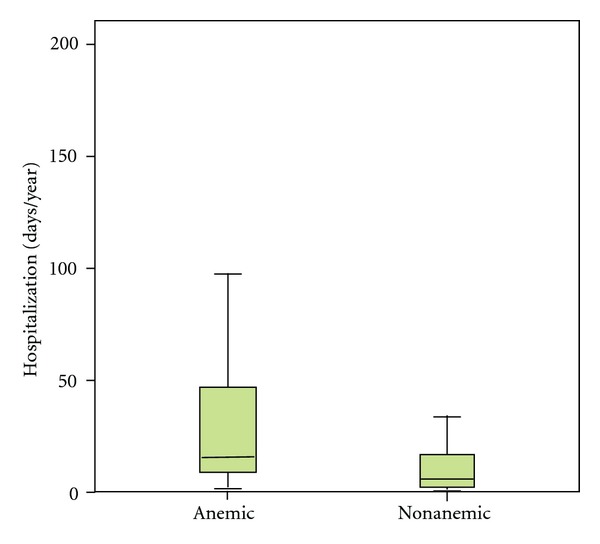
The hospitalization rate of anemic heart failure patients was significantly higher than in nonanemic patients (*P* < 0.05). Boxplots represent median and interquartiles. The whiskers extend to 1.5 times the difference between the first and third quartiles.

Anemic patients had significantly higher hospitalization rate from decompensated CHF than those without anemia (mean 35.1 ± 40.5 days per year versus 9.97 ± 9.65, *P* < 0.05) (Figure 2). Concerning the mortality rate of pediatric heart failure patients, there was no significant difference between anemic and nonanemic patients in this study population (23% versus 15%). 

## 4. Discussion

Although anemia is a commonly observed phenomenon in the setting of cardiac failure in children, its true prevalence is unknown and unpublished in the extant pediatric literature. In this retrospective study, 64% of DCM patients with congestive heart failure developed anemia. Anemia is prevalent in all age classes of DCM patients and without gender differences. The age of onset of anemia and severity are not depending. Less is known about the mechanism of anemia in pediatric congestive heart failure patients. In adult heart failure patients, several possible explanations for the high prevalence of anemia are known, and potential treatment options are reported.

Renal impairment is encountered in about 66% of heart failure patients, which may partly contribute to the lower than expected increase of erythropoietin in response to hypoxemia and decreased perfusion [1]. The use of angiotensin-converting-enzyme (ACE) the inhibitors also plays a role in causing anemia. Researchers have postulated that ACE inhibitors inhibit growth of erythroid precursors and may decrease erythropoietin production [7, 8]. ACE inhibitors are also routinely used in treatment of congestive heart failure of pediatric DCM patients. 

Hemodilution is common in congestive heart failure and found with an incidence of 46% in anemic CHF patients [9]. Furthermore, the anemia of inflammation, iron, vitamin B12, or folic acid deficiency is frequently reported in adult heart failure patients [1, 10]. 

In adult patients with CHF, anemia was found to be an independent risk factor for increased morbidity and mortality [3, 11]. In our study population, the hospitalization rate related to cardiac decompensation was investigated as a surrogate of morbidity. The hospitalization rate in patients developing anemia was significantly higher than in nonanemic patients (Figure 2). The potential mechanisms linking anemia to increased morbidity in CHF have not been characterized, but may be related to changes in ventricular loading conditions. Heart failure in congenital heart disease results from an excessive workload on the myocardium as a result of pressure or volume overload, with or without chronic cyanosis or myocardial dysfunction. Heart failure in cardiomyopathy results from inherited metabolic and muscle disorders [12]. The increased morbidity of anemic heart failure could be caused by destabilization of the hemodynamic situation of these patients. With regards to mortality, we found no significant difference between anemic and nonanemic patients. Eventually, larger study populations are necessary to reveal the influence of anemia on the mortality rate of pediatric DCM patients. 

Preliminary studies in adult CHF patients suggest the beneficial effects of treating anemia with erythropoietic agents, with regards to exercise capacity and quality of life [11]. Studies on pediatric patients are not reported, but anemia may be a potentially modifiable risk factor in pediatric DCM patients. 

The aim of this work was to determine the prevalence of anemia in children with heart failure and to evaluate the dependency on morbidity and mortality. Further studies investigating the cause of anemia in pediatric DCM patients and the possibility of reducing morbidity, hospitalization rate, and public health costs due to effective treatment of anemia are necessary and reasonable because of the high prevalence of anemia of all age classes.

**Limitations**

Limitations are those inherent to retrospective study. In the small study population, hemoglobin levels were checked at variable times but always concomitant to cardiac decompensation.

## 5. Conclusion

Anemia is prevalent in pediatric DCM patients of all age classes and is associated with a higher hospitalization rate corresponding to impaired heart failure symptoms as a surrogate for morbidity. Higher mortality did not appear in this population of DCM patients. Further studies are needed to determine the causes of anemia in pediatric heart failure patients and to explore whether correction of anemia could reduce morbidity and improve quality of life. 

## Figures and Tables

**Table 1:** Demographic and clinical characteristics of children with congestive heart failure due to DCM. (Values correspond to number (*n*) of patients or to means ± SD.)

	All patients *n* = 55	Anemic patients *n* = 35	Non-anemic patients *n* = 20	*P* value
Sex: male/female	28/27	19/16	9/11	n.s.
Mean age of first clinical heart failure symptoms (years)	4.06 ± 5.6	3.42 ± 5.3	5.19 ± 6.1	n.s.
Mean time of hospitalization (days per year)	25.94 ± 34.8	35.06 ± 40.48	9.97 ± 9.65	<0.05
Necessity of transfusion	17	17	0	<0.05
Death during followup	11	8	3	n.s.
